# Sexual Health and Men Who Have Sex with Men in Vietnam: An Integrated Approach to Preventive Health Care

**DOI:** 10.1155/2012/796192

**Published:** 2012-10-16

**Authors:** Le Minh Giang, Vu Duc Viet, Bui Thi Minh Hao

**Affiliations:** ^1^Department of Epidemiology, Hanoi Medical University, 1 Ton That Tung Street, Hanoi, Vietnam; ^2^Center for Research and Training on HIV/AIDS (CREATA), Hanoi Medical University, 1 Ton That Tung Street, Hanoi, Vietnam

## Abstract

*Background*. While HIV infection among men who have sex with men (MSM) in Vietnam has received increasing attention, most studies focus on HIV knowledge and established risk factors such as injection drug use. This paper proposes to address HIV risk among MSM from an integrated approach to preventive care that takes into account syndemic conditions such as substance use, mental health, and stigma, the latter of which prevents MSM from accessing health services. *Method*. Current studies related to MSM in Vietnam from 2000 onwards, gathered from peer-reviewed as well as non-peer-reviewed sources, were examined. *Results*. HIV and STI prevalence among MSM varied significantly by location, and yet HIV prevalence has increased significantly over the past few years. Most studies have focused on sexual risk behaviors, paying little attention to the broad spectrum of sexual health, including noninjecting drug use, heavy alcohol consumption, high rates of mental health distress and anxiety, and stigma. *Conclusion*. Future research and interventions targeting MSM in Vietnam should address their vulnerability to HIV from an integrated approach that pays attention to both sexual health and syndemic conditions.

## 1. Introduction

Research studies have shown that men who have sex with men (MSM) have unique health-care needs and that interventions focusing on this group should address these needs [1, 2]. MSM have been significantly affected by HIV epidemics all over the world. Research on MSM has found that the epidemics are reemerging in many wealthy countries and that many developing countries are paying more attention to the HIV epidemic among MSM [3]. A critical study on MSM in developing countries showed that the possibility of MSM being HIV infected was much higher than that of the general population [4]. In Asia, an association between HIV infection and drug use, including both injection and noninjection use, has been found [5]. However, non-injection drug use has been an increasingly important risk factor for HIV infection among MSM, whereas injecting drug use is thought to have a limited impact on the spread of HIV among this group [6]. Recreational drug use, especially the use of ecstasy and methamphetamines and alcohol use, is becoming increasingly common and is an important factors contributing to unprotected receptive anal intercourse [5, 7, 8]. The impact of substance use and myriad syndemic conditions has resulted in an alarming increase in HIV infection in Southeast Asia [9]. 

There are a number of studies on HIV infection among MSM in Vietnam, yet comprehensive understanding about sexual health, club drug use, and other syndemic conditions, such as mental health and stigma among MSM and how they relate to HIV vulnerability, is still not available. This study aims to identify gaps in understanding these issues in order to provide evidence supporting the call for an integrated approach to addressing HIV vulnerability and to improve preventive interventions targeting this at-risk group. The paper approaches this task by using two theoretical perspectives: the sexual heath model suggested by Robinson et al. [10, 11] and syndemic theory. 

The sexual heath model comprises 10 essential components of healthy human sexuality, such as talking about sex, culture and sexual identity, sexual anatomy and functioning, sexual health care and safer sex, challenges to sexual health, body image, masturbation and fantasy, positive sexuality, intimacy and relationships, and spirituality [10]. Many of these aspects are believed to influence an individual's ability to effectively reduce their HIV risk. The model assumes that people who are sexually healthy (i.e., those who are “sexually literate, comfortable, and competent”) are more likely to make healthy sexual choices, including choices related to HIV and sexual risk behavior [10]. On the other hand, syndemic theory refers to the concentration within a specific group of multiple cooccurring conditions that interact with and reinforce each other, ultimately giving rise to other health problems [12]. 

## 2. Method

The study reviewed the literature related to sexual health, substance abuse, and mental health among men who have sex with men in Vietnam from 2000 onwards. Literature included both articles published in peer-reviewed journals such as *Journal of Acquired Immune Deficiency Syndromes, AIDS and Behavior, AIDS Education and Prevention, Sexual Health, Culture, *and *Health and Sexuality, *as well as published research reports from institutions in Vietnam such as the Ministry of Health, Vietnam Administration of HIV/AIDS Control (VAAC), National Institute of Hygiene and Epidemiology (NIHE), Family Health International Vietnam, and United Nations Office on Drugs and Crime (UNODC) Vietnam. Key words and terms included men who have sex with men, MSM, gay men, homosexual men, HIV, sexually transmitted diseases (STDs), sexual transmitted infections (STIs), sexual health, sexual behavior, substance abuse, substance use, drug use, drug abuse, alcohol use, alcohol abuse, mental health, mental disorders, stigma, and Vietnam. As the number of extant studies designed only for MSM was limited, studies that have MSM as a subgroup and contain information about their sexual health, substance use, and mental health were also included. Articles and reports that were not in English were excluded to reduce misunderstanding due to translation.

## 3. Results and Discussion 

A total of 16 articles and reports addressing HIV/STI risk, sexual health, and substance use relating to MSM in Vietnam were identified and reviewed. Five articles were excluded, of which one was a duplication, three did not have information on MSM as a subgroup of their samples, and one was a review. Eventually, 11 studies were further reviewed; information about the articles is shown in Table 1. In only five studies were all of participants MSM. In the other studies, MSM constituted a subgroup of the research populations.

### 3.1. Dominant Focus on HIV and STI Risks

There were six articles investigating the prevalence of HIV among MSM in Vietnam. HIV prevalence rates among MSM varied among the cities studied and even varied by survey for individual cities. For instance, a study in 2005 found that HIV prevalence among 295 MSM in rural settings in Khanh Hoa province was 0% [13]. Another study in 2004 showed that HIV prevalence among MSM in Ho Chi Minh City (HCMC) was 8% [14], but the HIV/STIs Integrated Biological and Behavioral Surveillance (IBBS) in 2006 revealed that the figure for MSM in HCMC was 5.3% [15]. However, it is obvious that the HIV infection among MSM was increasingly significantly during that time. For instance, HIV prevalence in Hanoi increased from 9.4% in 2006 to 17.4% in 2009, while the figures for HCMC were 5.3% and 16.7%, respectively [15, 16]. Besides Hanoi and HCMC, HIV prevalence in some other large cities was also observed: for Hai Phong it was 15% for MSM trading sex for money and 17% for those who were not sex traders, whereas for the city of Can Tho, the figure was 9% and 5%, respectively [16]. The increased HIV acquisition in Vietnam is in accordance with the HIV epidemic trend among MSM in Asia [16]. 

STI prevalence was investigated in four studies focusing on syphilis, chlamydia, gonorrhea, and/or HPV. According to findings from the IBBS surveys [16], for every five MSM in HCMC, one was infected with at least one of three sexually transmitted infections (syphilis, chlamydia, or gonorrhea). The figure for Hanoi was 19% of MSM trading sex for money and 13% of MSM having sex without receiving money. In comparison to 2006, the picture of STIs was the opposite for Hanoi and HCMC. The prevalence in Hanoi decreased in both groups, those having sex for money and those without receiving money, whereas in HCM, it increased in both groups.

Studies have shown that MSM in Vietnam have been at increased risk for HIV infection [14, 17], and that HIV infection has been associated with number of sex partners and selling sex [14, 18]. Nguyen et al. [14] found that MSM with more than five male sex partners (OR = 2.43; 95% CI, 1.14–5.17) and selling sex (OR = 8.61, 95% CI, 1.20–61.6) had a higher chance of HIV infection, whereas Colby [18] showed that, on average, HIV-negative MSM had about 9 sexual partners in the past year, whereas HIV-positive MSM had 14 partners.

 Together with HIV acquisition, the association between STIs and sexual behavior is a concern in research on MSM. The IBBS survey [16] showed that except for the city of Can Tho, where STI prevalence was 5% for MSM who did not receive money for sex and 9% for MSM receiving money for sex, the STI prevalences in other cities were all above 10%. High prevalence of STIs could be explained by unsafe sex. The IBBS 2009 report also revealed that the proportion of MSM who use condom was low, under 50% in all cities, whereas the proportion of condom use among MSM in HCMC reduced when comparing to that in 2006 [16]. In another study, 33% of MSM reported unprotected anal intercourse (UAI) within the past month, with 21% reporting UAI with male clients [18]. Unprotected anal intercourse may not be the only risk factor; research also indicates that anal STIs can also be transmitted via sexual partners' fingers or tongue [19].

The proportion of consistent use of lubricants for anal sex was low in the studies reviewed. Nguyen et al. [14] reported that only 44% of MSM always used lubricant during anal sex in the past 6 months. According to the study, saliva was the most frequently used lubricant, accounting for 53% of MSM, whereas other lubricants were antibiotic ointment, skin lotion, and lubricants (water soluble and nonsoluble). Although there was no research on the effect of using saliva as a lubricant in Vietnam, the use of saliva could result in infection by a salivary pathogen such as the herpes virus, hepatitis B virus, and cytomegalovirus [20].

 As research showed that MSM, especially MSM selling sex, were an HIV bridge group who might have unprotected sex with individuals who are at low risk of HIV exposure [14], current studies also explored HIV risk among male sex workers (MSWs) [18, 21]. MSWs reported that they had about 10 male sex partners during the past 30 days, and that they did not always use condoms when having anal sex. About 30% of MSWs in Hanoi, 25% in HCMC, and 55% in Nha Trang reported having unprotected anal intercourse [21].

### 3.2. Lack of Attention to Sexual Health and Syndemic Conditions

Sexual health is defined as a state of physical, emotional, mental, and social well-being concerning sexuality, as opposed to only referring to the absence of disease, dysfunction, or infirmity [10]. It entails considering sexuality and sexual relationships in a positive and respectful manner, and the possibility of safe as well as pleasurable sexual experiences [11]. The sexual health model suggested by Robinson et al. [10] is a broad approach to HIV prevention. 

 Sexual orientation has received more attention in current studies than in the past. Though public opinion regarding MSM is more open in Vietnam at this time [22], the proportion of MSM who do not disclose their sexual orientation and consider themselves as “bong kin” (the Vietnamese term of “bong kin” refers to men who do not want to be identified as same-sex attracted men and exhibit their masculinity appearance), a nontransvestite, or homosexual, is still very high. For instance, 66% of MSM in HCMC considered themselves as homosexual [17] and up to 77% of them were non-transvestites [14]. A challenging issue in studying male homosexual identities is that there is no common agreement or an official guide on categorizing sexual orientations, making comparisons among studies impossible. 

Studies of cooccurring psychosocial problems, or psychosocial “syndemics,” have found that mental health problems among MSM are associated with HIV risk, and that there is an additive risk with each psychological problem with respect to sexual risk-taking behavior in MSM [23]. As MSM suffer a greater number of psychological problems, their risk for engaging in sexual risk behaviors grows, as does their risk for HIV infection [23]. Mustanski et al. [24] found synergistic effects of multiple psychological risk factors on sexual risk taking in young HIV-negative MSM. Recent research suggests that this phenomenon may also extend to HIV-infected MSM. In a sample of 380 HIV-infected MSM, those with one to three syndemic indicators (childhood sexual abuse, PTSD, anxiety disorders, depression, poly-substance use, alcohol abuse) had a greater than twofold increase in the likelihood of exhibiting sexual transmission risk behavior, whereas those with four or more syndemic indicators experienced a fourfold increase in such behavior [25].

In Vietnam, substance abuse, especially involving alcohol and amphetamines, and mental health problems are common among MSWs and are associated with unsafe sex [18]. Analysis of six syndemic conditions (alcohol use, amphetamine use, suicide risk, low self-esteem, PTSD score, childhood sexual abuse) and unprotected anal intercourse (UAI) showed that when the number of syndemic conditions was high, the possibility of UAI was high; the percentages of those having four, five, or all six conditions also reporting UAI were 50%, 67%, and 100%, respectively [18]. 

The following are various conditions that call for broader attention. 

#### 3.2.1. Use of a Broad Range of Drugs

A paper reporting the presentation of Grant Colfax, M.D., (San Francisco Department of Public Health) showed that prevalence of substance use among MSM continues to be high and that there is an association between noninjection drug use with HIV risk [26]. Some theories explaining this association are that altered mental states lead to reductions in condom use, and enhanced desire/pleasure and decreased pain lead to more partners, longer sex, and tissue damage/blood contact [26]. A review of studies on the association between club drugs and HIV risks proposes that such drugs result in a number of impacts on the human body, such as changed mental state, decreased experience of pain, and enhanced sexual function. This leads to reduced condom use, tissue damage or increased bleeding, and an increased number of sexual partners, all of which increase the risk of STI/HIV infection [27]. 

In Vietnam, there is a strong association between drug injection and HIV infection among men who have sex with men [14, 15, 28]. There is also an association between using drugs and selling sex [29]. However, knowledge about substance use and its association with risk behaviors for HIV and other health problems among MSM in Vietnam is limited to data collected from cross-sectional and opportunistic samples [17]. Notably, while injection drug use among MSM seems to be unchanged, HIV infection among this population sharply increased in Hanoi and HCMC from 2005 to 2009 [15, 28]. It is suggested that new dynamics of HIV risk may occur among MSM and that drug use may be associated with those changes, which requires further investigation. 

Drug use among MSM, particularly non-injection drug use, has become more common. In 2001, fewer than 2% of MSM in HCMC admitted to using intravenous drugs [17]. Another study in HCMC in 2004 reported that 6% of the MSM had “ever used drugs,” both injection and non-injection, of whom 66% had used heroin, 4% opium, 4% amphetamine, and 25% tranquilizers [14]. The results of IBBS surveys in 2006 and 2009 showed that the proportion of MSM who had ever used drugs increased from 22.8% in Hanoi and 21.0% in HCMC in 2006 to 31.8% and 25.3% in 2009, respectively. However, the proportion of those who had ever injected drugs remained stable (9.2% in Hanoi and 3.8% in HCMC in 2006 and 6.0% and 8.0% in 2009, resp.) [15, 16].

Currently, research on substance use among MSM has paid more attention to non-injection drugs, or so-called “club drugs.” A subsample of men selling sex, as part of a larger study of young heroin users in Hanoi in 2002, was analyzed. In addition to heroin, many men selling sex reported the use of other types of drugs in the past 30 days: 13% used marijuana, 13% used amphetamine/methamphetamine, 8% used ecstasy (MDMA), 6.3% used opium, and 3.8% used morphine [30]. Research in HCMC in 2010 revealed that 27% of MSM reported ecstasy use, whereas only 2% reported injecting heroin use [17]. Research showed that, partly due to the misconception that the use of club drugs such as ecstasy and “ice” (methamphetamine) would help them quit heroin, drug use was shifting from heroin to club drugs as well as polydrug use [29].

 In 2012, a report on amphetamine-type stimulants (ATSs) in Vietnam was published [31]. It included information about knowledge and use of ATSs among MSM. According to the report, the percentage of MSM who had heard of methamphetamine, ice, and ecstasy was 36%, 70% and 96%, respectively. Knowledge of the effects of ATSs was low with regard to depression (51%), decreased appetite (72%), violent or uncontrolled behavior (71%), and increased chance of sexual risk behavior (75%). MSM on average knew 11 people in their social network who used ATSs. The percentage of ATSs use was 11% for methamphetamine, 82% for ecstasy, and 57% for ice. Frequency of use of ecstasy was 10% for several times per week, 17% for once per week, 46% for several times per month, and 28% for once or twice in last 90 days. Frequency of use of ice was 21% for several times per week, 21% for once per week, 44% for several times per month, and 15% for once or twice in the last 90 days. 

It is documented that unsafe sex is associated with drug use [29]. Some MSM combine drugs; for example, ketamine or marijuana is combined with ecstasy to enhance sexual pleasure. Moreover, MSM fail to use condoms during sex due to loss of control under the influence of drugs. MSM would consciously use a condom when having sex while not drunk, but did not think of using a condom while high on ecstasy or ice. Condom use among MSM may be limited during group sex when drugs are involved. In addition, there is a clear link between drug use and sex work; drugs are used as a tool for sex work, specifically to enhance sexual performance and to increase sexual confidence with male clients. Selling sex for drugs may be the most desperate option and is the most risky situation due to it leading to accepting unprotected sex by all means to have drugs [29]. However, a limitation of studies on MSM is that they do not show a statistically significant association between non-injection drug use and risk behavior for HIV infection.

#### 3.2.2. Heavy Alcohol Use 

The link between alcohol use and risky sexual behavior is investigated in a number of studies. Whereas one study found that alcohol use is a factor related to unprotected sexual behavior [32], another study found little evidence for a direct connection between alcohol use and risky sex [33]. On the other hand, several studies suggested that outcome expectancies and sensation seeking might play a role in predicting both unsafe sex and alcohol use. A study on HIV-positive MSM stated that alcohol use plays a role in three basic sexual scripts, namely, “routine,” “spontaneous,” and “taboo,” which have their own sources of risk for unsafe sex [34]. Sexual scripts are the narrative ways in which people organize their beliefs and expectations regarding sexual behaviors [34]. In routine sexual scripts, participants drank alcohol in a planned and conscious manner, and used it “as a social lubricant and as a prerequisite for sex.” In spontaneous sexual scripts, people “ascribed their sexual activities or their partner-selection choices to the effects of alcohol.” On the other hand, in taboo scripts, participants “engaged in sexual behavior and/or thoughts that they felt were socially stigmatized” while under the influence of alcohol [34].

In a study in Ho Chi Minh City, the percentage of alcohol drinking among MSWs was high (66%), of which 19% of them drank heavily (weekly binges) [18]. Another study showed a high prevalence of alcohol use at the last sexual encounter with Vietnamese clients: 41%, 34%, and 64% for Hanoi, HCMC, and Nha Trang, respectively. The prevalence of alcohol use at last sexual encounter with non-Vietnamese clients was 21%, 20%, and 50% [21]. 

#### 3.2.3. Burden of Mental Health Disorders

Studies worldwide have found that MSM are disproportionally affected by mental health problems [35] and that sexual minorities, MSM included, are at increased risk for depressive, anxiety, and substance use disorders [36–38]. In Vietnam, a current study of MSM showed that their knowledge about the mental health effects of ATSs is limited [31]. While MSM believed that the use of ATSs could lead to hallucinations, the majority of them did not seem to think that the use of ATSs could lead to short- or long-term feelings of depression. This finding was more pronounced regarding the use of ecstasy than for ice. MSM were more likely to think that using ice could lead to feelings of depression, although this perception was still under 50%. Another study on MSWs revealed a high prevalence of mental health disorders [21]. Percentages of significant psychological distress (≥16) among MSM were 61% in Hanoi, 49% in HCMC, and 71% in Nha Trang, while the figures for moderate-to-high anxiety were 17%, 11%, and 30%, respectively. The study also suggested a possible association among alcohol use, drug use, and sexual risk. The fact that data on mental health disorders is limited reflects the lack of research on mental health among MSM in Vietnam.

#### 3.2.4. Persisting Stigma and Limited Access to Services

Historical and cultural norms may prove to be significant obstacles to any HIV/AIDS prevention efforts aimed at MSM in Vietnam. Stigma related to homophobia has been fueled by misconceptions of homosexuality [39]. Criminalization of homosexuality can exacerbate HIV epidemics [40]. In Vietnam, fear and misperceptions about the risks and routes of HIV transmission were considered as principle causes of stigma and discrimination among people living with HIV/AIDS [39]. Discrimination in health care settings was common. Manifestations included nonverbal actions, such as being ignored or stared at with disapproving facial expressions, and being treated with an unfriendly attitude. Verbal abuse and routine service refusal were also observed. MSM felt that they faced double stigmatization due to their homosexuality and their HIV infection. Many MSM said that the only way to cope with the discrimination they faced from neighbors was to ignore it [41, 42]. 

A study in Ho Chi Minh City showed that the percentage of MSWs who had access to services increased significantly from 2009 to 2010. For instance, the percentage of MSWs receiving condoms increased from 45% to 74%, lubricant from 30% to 71%, HIV brochures for MSM from 25% to 67%, and HIV tests from 37% to 50%. However, the proportion of MSWs receiving all four services was still low, accounting for only 34%. In 2010, only 36% of MSWs received STI exams, whereas 68% met with peer educators [18]. Moreover, MSWs rarely disclosed their sexual contacts with men to health care staff due to stigma and self-stigma, which led to ineffective engagement with health services [21]. 

## 4. Conclusion

Although this study was conducted in a rigorous manner, it still has some limitations. As the number of quantitative studies, as well as the size of samples, was limited, data were not sufficient for a meta-analysis, reducing the power and generalization of the results. The use of qualitative studies partly compensated for this drawback, yet solid arguments were not always gathered. Lastly, although statistics reported in government reports were largely not peer-reviewed, in the context of the limited number of studies on MSM, using these reports was a reasonable choice. 

This paper shows that a limited number of studies have addressed a number of major aspects related to the broad syndemic of health problems among men who have sex with men in Vietnam. And yet, knowledge on various health risks among MSM in Vietnam was incomplete, while the relationship among sexual health, drug, and alcohol use, and health-related problems was multifaceted. Among various health risks, HIV and STIs, as well as drug use, have received more attention, while other conditions, such as alcohol use, mental health, and stigma, have received much less attention. Despite this lack of knowledge, enough evidence exists to support the call for an integrated approach to addressing HIV infection among this group. Future research should focus on the sexual health model [10] and syndemic conditions that prevent MSM from achieving better health. Further research also should focus on these syndemic conditions to provide evidence for better preventive initiatives. 

## Figures and Tables

**Table 1:** Research studies on men who have sex with men (MSM) included in this paper.

Study number	Authors publish year	Year	Location	Participants	Sample size
1	Colby 2010 [18]	2010	Ho Chi Minh City	MSWs* (are MSM)	300 (300)
2	Nguyen et al, 2008 [14]	2004	Ho Chi Minh City	MSM	600
3	CREATA 2012 [21]	2009	Hanoi	MSWs (are MSM)	710
			Ho Chi Minh City		
		2011	Nha Trang		
4	Colby 2003 [17]	2001	Ho Chi Minh City	MSM	219
5	UNODC 2012 [31]	2010		Risk groups (incl. MSM)	1352 (270)
		2011			
6	NIHE and FHI [15]	2006	Hanoi	Risk groups (incl. MSM)	(790)
			Ho Chi Minh City		
7	MOH and NIHE [16]	2009	Hanoi, Haiphong	Risk groups (incl. MSM)	(1596)
			Ho Chi Minh City Cantho		
8	Colby et al. 2008 [13]	2005	Khanh Hoa	MSM	295
9	Vu et al. 2012 [29]	2009	Hanoi, Ho Chi Minh City	MSM, MSWs, transgender, (stakeholders)	115 (9)
10	Vu et al. 2008 [41]	2004	Ho Chi Minh City	MSM (key informants)	90 (16)
11	Ngo et al. 2009 [22]	2007	Hanoi,	Young men and MSM	NA
		2008	Ho Chi Minh City		

*“MSWs” stands for “male sex workers.”
